# Supplementary material for: Content-rich biological network constructed by mining PubMed abstracts
Source: BMC Bioinformatics. 2004 Oct 8;5:147. doi: 10.1186/1471-2105-5-147 (PMC528731; doi:10.1186/1471-2105-5-147)
Supplement: Additional File 2 — The original results of the above study (non-essential files are deleted to keep the file size under the limit set by BMC bioinformatics). [file 1471-2105-5-147-S2.bz2 › chilibotAdditionalFile2/dip50/753ID9852089E18138/html/DNAA_DNAB.html]

 


 **DNAA** and **DNAB** 
  
Found 99 abstracts in PubMed, retrieved 50.  
 

 What does Google say? 
 PDF only 
| .edu only 

---

**Interactive relationship** (e.g. stimulation, inhibition, etc)

**Neutral relationship**- On the analysis of this process, the antibody appears to interfere with the physical interaction between  **DnaA**  and  **DnaB**  protein in the  **DnaB** .DnaC complex.  Ref: 8106460 J Biol Chem, 1994
- The interaction domains of the  **DnaA**  and  **DnaB**  replication proteins of Escherichia coli.  Ref: 10972842 Mol Microbiol, 2000
- Escherichia coli  **DnaA**  protein loads a single  **DnaB**  helicase at a  **DnaA**  box hairpin.  Ref: 12161435 J Biol Chem, 2002
- We propose that contacts between  **DnaA**  and  **DnaB**  occur via two interaction sites on each of the proteins.  Ref: 10972842 Mol Microbiol, 2000
- **DnaA**  protein directs the binding of  **DnaB**  protein in initiation of DNA replication in Escherichia coli.  Ref: 8106460 J Biol Chem, 1994
- Both  **DnaA**  and TrfA proteins are required for  **DnaB**  induced template unwinding.  Ref: 9407123 J Biol Chem, 1997
- We show that  **DnaA**  protein actively mediates the entry of  **DnaB**  at oriC.  Ref: 9852089 J Biol Chem, 1998
- Other evidence suggests that proline 28 of  **DnaA**  is involved in the recruitment of  **DnaB**  to oriC.  Ref: 12864864 Mol Microbiol, 2003
- Although the  **DnaA**  protein directs the  **DnaB**  DnaC complex to the plasmid replication origin, it cannot by itself activate the helicase.  Ref: 9407123 J Biol Chem, 1997
- With this system, a specific interaction between the  **DnaA**  and  **DnaB**  proteins is required for delivery of the helicase to the RK2 origin region.  Ref: 9407123 J Biol Chem, 1997
- Domains of  **DnaA**  protein involved in interaction with  **DnaB**  protein, and in unwinding the Escherichia coli chromosomal origin.  Ref: 8702501 J Biol Chem, 1996
- These studies suggest regions of  **DnaA**  protein involved in interaction with  **DnaB**  protein, and in unwinding of oriC, or low affinity binding of ATP.  Ref: 8702501 J Biol Chem, 1996
- We defined physical contacts between  **DnaA**  and  **DnaB**  that involve residues 24 86 and 130 148 of  **DnaA**  and residues 154 210 and 1 156 of  **DnaB**  respectively.  Ref: 10972842 Mol Microbiol, 2000
- These results provide the first direct evidence that one of the roles of  **DnaA**  protein is to act as a site for binding of  **DnaB**  protein to the DNA and perhaps orients  **DnaB**  helicase to account for the directionality of replication fork movement.  Ref: 8106460 J Biol Chem, 1994
- Size exclusion chromatography, surface plasmon resonance, and electron microscopy experiments demonstrated that the  **DnaB**  helicase is delivered to the  **DnaA**  box region, which is localized approximately 200 base pairs upstream from the region of origin opening and a potential site for helicase entry.  Ref: 11316803 J Biol Chem, 2001
- **DnaA**  was distributed throughout the cytoplasm, but both  **DnaB**  and DnaI were always detected as foci during the cell division cycle.  Ref: 10844689 Mol Microbiol, 2000
- the  **DnaA**  boxes must be on the correct side of the helix to load  **DnaB**  helicase.  Ref: 9525957 J Biol Chem, 1998
- A novel priming system employing  **dnaA** ,  **dnaB** , dnaC, and primase on a hairpin containing a  **dnaA**  box sequence.  Ref: 2168401 J Biol Chem, 1990
- We suggest that the roles of  **DnaA**  in P1 plasmid and bacterial replication are similar origin opening and loading of the  **DnaB**  helicase.  Ref: 8223464 EMBO J, 1993
- That only two  **DnaB**  hexamers are bound, one for each replication fork, suggests that the binding of additional molecules of  **DnaA**  in forming the prepriming complex restricts the loading of additional  **DnaB**  hexamers that can bind at oriC.  Ref: 11551962 J Biol Chem, 2001
- The ABC primosome is assembled from  **DnaA** ,  **DnaB**  and DnaC proteins at a stem and loop structure containing a  **dnaA**  box within its stem A site, and catalyses primer RNA synthesis for DNA chain elongation.  Ref: 7607206 Eur J Biochem, 1995
- **dnaA**  protein specifically recognizes and binds the single stranded hairpin and permits the loading of  **dnaB**  protein to form a prepriming protein complex containing  **dnaA**  and  **dnaB**  proteins which can be physically isolated.  Ref: 2168401 J Biol Chem, 1990
- Taking into account our recent finding that transcriptional activation of ori lambda seems to be  **dnaA**  regulated to be published elsewhere, we suggest that the  **DnaA**  lambda Pts1 incompatibility occurs at the insertion of the ori lambda bound lambda O lambda P  **DnaB**  preprimosome between the complementary lambda DNA strands.  Ref: 7789753 Genetics, 1995
- The introduction into the plasmid of a hairpin structure, whose stem carries a  **DnaA**  box, mediates conversion of SS c into dsDNA and makes plasmid replication independent of the B. subtilis  **dnaB**  function.  Ref: 8039651 FEMS Microbiol Lett, 1994
- The addition of  **DnaA**  extends melting to the AT rich region, which could then serve as the entry site for the  **DnaB**   **DnaA**  complex, much as has been documented for oriC dependent replication.  Ref: 9003285 Mol Gen Genet, 1996
- The initiation of chromosome replication in Escherichia coli requires the recruitment of the replicative helicase  **DnaB**  from the DnaBC complex to the unwound region within the replication origin oriC, supported by the oriC bound initiator protein  **DnaA** .  Ref: 10972842 Mol Microbiol, 2000
- The  **dnaA**  initiator protein binds specific sequences in the 245 base pair Escherichia coli origin oriC to form a series of complexes which eventually are opened enough to admit  **dnaB**  helicase into a prepriming complex Bramhill, D.  Ref: 2153124 J Biol Chem, 1990
- Experiments using dnaC and  **dnaB**  ts strains suggest that  **DnaA**  control of fts transcription may be indirect, at least in part, with fts responding to the rate of initiation at oriC as well as to changes in  **DnaA**  protein level per se.  Ref: 2546042 Mol Gen Genet, 1989
- The  **DnaA**  replication initiation protein has been shown to be essential for DNA strand opening at the AT rich region of the replication origin of the Escherichia coli chromosome as well as serving to recruit and position the  **DnaB**  replicative helicase at this open region.  Ref: 12835421 Proc Natl Acad Sci U S A< MedlineTA>Proc Natl Acad Sci U S A, 1989

**Non-interactive relationship** (e.g. studied together, co-existance, homology, etc.)

- RepA protein of pPS10 promotes in vitro plasmid replication in extracts from E. coli, and this replication depends on host proteins  **DnaA** ,  **DnaB** , DnaG, and SSB.  Ref: 7635822 J Bacteriol, 1995
- Similar stabilization of O2 Co II Blm bound to  **DNAa**  but not to  **DNAb**  was also observed by ESR spectroscopy.  Ref: 9100034 Biochemistry, 1997
- In the prepriming complex, the stoichiometries are 10  **DnaA**  monomers and 2  **DnaB**  hexamers per oriC plasmid.  Ref: 11551962 J Biol Chem, 2001
- Kid inhibits in vitro replication of CoEl to a basal level without altering the superhelicity of the template but it does not substantially affect in vitro replication of P4, a  **DnaA** ,  **DnaB** , DnaC and DnaG independent replicon.  Ref: 7723014 J Mol Biol, 1995
- A priming mechanism requiring  **dnaA** ,  **dnaB** , and dnaC proteins operates on a single stranded DNA coated with single stranded DNA binding protein.  Ref: 2168401 J Biol Chem, 1990
- Thus, DnaAcos protein basically sustains affinity to a  **DnaA**  binding sequence and functions in the loading of  **DnaB**  helicase onto single stranded DNA.  Ref: 8825085 Mol Microbiol, 1995
- The Escherichia coli PriA protein loads the  **DnaB**  replicative helicase at branched DNA structures independently of the replication initiator protein,  **DnaA** , and thereby facilitates assembly of functional replisomes at sites removed from oriC.  Ref: 12581361 Mol Microbiol, 2003
- Cross linking studies confirm that  **DnaA**  and  **DnaB**  proteins interact directly.  Ref: 8106460 J Biol Chem, 1994
- Initiation of SPP1 replication is independent of the host  **DnaA**  replisome organizer,  **DnaB** , DnaC and DnaI products, but requires the DnaG DNA primase and the DNA gyrase.  Ref: 8126723 J Mol Biol, 1994
- However, as shown in this study, TrfA 44 specifically interacts with the  **DnaB**  replicative helicase of Pseudomonas Pseudomonas putida and Pseudomonas aeruginosa and initiates the formation of a prepriming open complex in the absence of  **DnaA**  protein.  Ref: 12835421 Proc Natl Acad Sci U S A< MedlineTA>Proc Natl Acad Sci U S A, 1994
- Stoichiometry of  **DnaA**  and  **DnaB**  protein in initiation at the Escherichia coli chromosomal origin.  Ref: 11551962 J Biol Chem, 2001
- Although the origin opening caused by binding of the host  **DnaA**  protein has been shown to be critical for the loading of the  **DnaB**  helicase, to date there has been no direct evidence presented for the formation of the  **DnaB**  complex at the  **DnaA**  box site.  Ref: 11316803 J Biol Chem, 2001
- High levels of  **DnaA**  protein in the presence of the TrfA protein also resulted in the stimulation of open complex formation and  **DnaB**  helicase loading on oriV, even in the absence of the four  **DnaA**  boxes.  Ref: 10417640 Mol Microbiol, 1999
- As was observed previously for  **DnaA** , overexpression of the wild type  **DnaB**  protein conversely stabilized the inheritance of partition defective plasmids while not increasing plasmid copy number.  Ref: 10601213 J Bacteriol, 1999
- Although, interaction of  **DnaA**  with  **DnaB**  could not substitute for RepA  **DnaB**  interaction for helicase loading,  **DnaA**  along with integration host factor, DnaC, and RepA was essential for helicase loading.  Ref: 9874774 Proc Natl Acad Sci U S A, 1999
- The bacterial RNA polymerase, the initiation protein  **DnaA** , and the primosomal proteins  **DnaB** , DnaC, DnaG and DnaT are not required.  Ref: 1851552 Nucleic Acids Res, 1991
- Thus, the TrfA 44 initiation protein has the multifunctional properties of recruiting and positioning an active form of the  **DnaB**  helicase at the RK2 replication origin by a  **DnaA**  independent process.  Ref: 12835421 Proc Natl Acad Sci U S A< MedlineTA>Proc Natl Acad Sci U S A, 1991
- In contrast to other DNA damage inducible replication genes, such as  **dnaA** , dnaN, dnaQ, and polA, expression of which is not inducible in recA and lexA mutants, the induction of  **dnaB**  was also observed in a recA1 mutant.  Ref: 7476872 Mol Gen Genet, 1995
- iii Mutations in either the  **dnaA** ,  **dnaB** , dnaC, dnaE, dnaG, or ssb gene, when introduced into a strain containing a bioA lac fusion, produced a blue colony color without an increase in beta galactosidase synthesis.  Ref: 2142938 J Bacteriol, 1990
- It differs in this respect from  **dnaA**  gene expression which is unaffected when initiation of replication is inhibited by  **DnaB**  or DnaC inactivation.  Ref: 2546042 Mol Gen Genet, 1989
- We report here that the Escherichia coli replication proteins  **DnaA** , which is required to initiate replication of both the chromosome and plasmid pSC101, and  **DnaB** , the helicase that unwinds strands during DNA replication, have effects on plasmid partitioning that are distinct from their functions in promoting plasmid DNA replication.  Ref: 10601213 J Bacteriol, 1999
- Co and Fe bleomycins Blms have been reacted with  **DNAa** , d GGAAGCTTCC 2, containing a specific site for cleavage, and  **DNAb** , d GGAAATTTCC 2, a closely related nonspecific 10 mer, to survey whether features of structure and reactivity of these adducts vary systematically as a function of the base sequence of the DNA oligomer.  Ref: 9100034 Biochemistry, 1997
- Since  **DnaA**  induces a local unwinding of oriC, one model is that the limited availability of single stranded DNA at oriC restricts the number of  **DnaB**  molecules that can bind.  Ref: 12161435 J Biol Chem, 2002
- At non permissive temperature, plasmid replication depends on the  **dnaB** , dnaC, dnaG and dnaI initiation replication products, but does not require the  **dnaA**  function.  Ref: 8339905 FEMS Microbiol Lett, 1993
- In E.coli DnaT and DnaC are responsible for loading  **DnaB**  onto the DNA during primosome assembly, in the PriA and  **DnaA**  mediated cascades, respectively.  Ref: 11842108 Nucleic Acids Res, 2002
- DNA polymerase III and two initiation proteins,  **DnaA**  and  **DnaB** , were required for in vitro replication as observed in vivo.  Ref: 8065264 Mol Microbiol, 1994
- It has previously been shown that the reaction required the E. coli  **DnaA**  initiator protein, the  **DnaB**  helicase, DnaC protein, RNA polymerase, and DNA gyrase.  Ref: 2181445 Proc Natl Acad Sci U S A, 1990
- This is based on the competition between the lambda P protein and the host  **DnaA**  and DnaC proteins for  **DnaB**  helicase.  Ref: 11429468 Microbiology, 2001
- The AT rich region could be unwound by E. coli  **DnaA**  protein, and the  **DnaB**  helicase was loaded into the single stranded bubble.  Ref: 11606418 EMBO Rep, 2001
- As in the case of several Gram positive bacteria, essential genes involved in basic cellular functions, such as DNA or RNA metabolism  **dnaA** ,  **dnaB** , dnaN, gyrB, gyrA, pcnB, recF, rnpA, ssb, cell wall synthesis ponA, pbpA and probably cell division gidB, rodA were found.  Ref: 8969512 Microbiology, 1996
- Strand specific loading of  **DnaB**  helicase by  **DnaA**  to a substrate mimicking unwound oriC.  Ref: 12421318 Mol Microbiol, 2002
- In keeping with this result, the isolated preprimosome, which appears to contain  **DnaA**  and  **DnaB**  proteins, is capable of hydrolyzing dATP as well as ATP and GTP.  Ref: 7607206 Eur J Biochem, 1995
- One can also conclude that the  **DnaA**  regulated transcriptional activation of ori lambda acts at the step, in which all these gene products cooperate, i.e. during preprimosome loading and chaperone mediated release of  **DnaB**  from P protein inhibition.  Ref: 8914519 Mol Gen Genet, 1996
- The initiation of replication from oriV RSF1010, the replication origin of the broad host range plasmid RSF1010, depends on RepA helicase, RepB primase, and RepC initiator protein, encoded by RSF1010 itself, while this initiation event in E. coli is independent of  **dnaA** ,  **dnaB** , dnaC, and dnaG Scherzinger et al.  Ref: 8690079 FEBS Lett, 1996
